# Assessing the impact of co-created community initiatives on health and equity: a protocol for population-based surveys within the CAIR project in Elche, Spain

**DOI:** 10.3389/fpubh.2026.1766000

**Published:** 2026-06-03

**Authors:** Antonio Moreno-Llamas, Noelia Navarro-Aranda, María José Sanchís-Ramón, Elisa Chilet-Rosell, Blanca Lumbreras, Clara Blanes-Mira, Elsa López Pintor, Ildefonso Hernández-Aguado, Lucy Anne Parker

**Affiliations:** 1 Instituto de Investigación en Biotecnología y Salud (IDIBE), Universidad Miguel Hernández (UMH), Avenida de la Universidad, Elche, Spain; 2 Departamento de Salud Pública, Historia de la Ciencia y Ginecología, Universidad Miguel Hernández (UMH), Alicante, Spain; 3 Department of Sociology and Social Work, University of Basque Country (UPV/EHU), Leioa, Spain; 4 Research Group Social Determinants of Health and Demographic Change-OPIK, Leioa, Spain; 5 Consorcio de Investigación Biomédica en Red de Epidemiología y Salud Pública (CIBERESP), Madrid, Spain; 6 Departamento de Ingeniería, Área de Farmacia y Tecnología Farmacéutica, Universidad Miguel Hernández, Alicante, Spain

**Keywords:** antibiotic use, co-creation, health literacy, health-related quality of life, social capital, Spain

## Abstract

**Introduction:**

The Western health system has primarily focused on a biomedical approach, prioritizing pharmaceutical and clinical treatments while overlooking the social, economic, psychological, and environmental dimensions of health. As a result, biomedical solutions have received disproportionate attention, while non-pharmacological, community-based interventions remain underutilized. The CAIR project seeks to address this imbalance by co-creating community-driven health initiatives in Municipal District 2 of Elche, Spain. This protocol describes the methodology for two population-based surveys conducted before and after the co-creation process to assess changes in key health and wellbeing indicators at the community level.

**Methods:**

A community-based participatory process will be implemented to engage residents in co-creating initiatives to promote health and wellbeing over a 3-year period (2026–2028). To evaluate population-level changes related to this co-creation process, a representative survey of 600 adult residents (≥18 years) in Municipal District 2 of Elche (Spain) will be conducted using stratified random sampling, administered both before and after the co-creation process. The survey will assess a set of selected indicators: health-related quality of life, critical health literacy, community capital, social capital and cohesion, and knowledge, attitudes, and practices related to antibiotic use. Sociodemographic data will also be collected to enable equity-focused analyses, including both relative and absolute differences between population groups. Findings from the baseline survey will inform the design and implementation of the co-created initiatives, particularly by highlighting equity considerations across population groups, while a before–after comparisons of survey indicators will be used to explore the effect of the co-created initiatives.

**Discussion:**

Overall, this study will generate population-level evidence on the effect of co-created initiatives in a low-income, culturally diverse urban setting in Spain. By documenting the contribution of co-creation to improve key health and wellbeing indicators, it will provide the evidence needed to further advance participatory approaches in public health.

**Trial registration:**

https://clinicaltrials.gov/ct2/show/NCT07237971, Research Registry Unique Identifying Number: NCT07237971.

## 1 Introduction

The predominant approach in the Western health system continues focusing on a biomedical perspective on health, emphasizing individual-level solutions such as the application of drugs and treatments. This biomedical model overlooks the broader social, economic and environmental factors that influence health and wellbeing (1). It has also contributed to a growing trend toward medicalisation, in which health problems are interpreted as medical issues, ignoring their social and psychological dimensions (2). Moreover, biomedical solutions have received disproportionate attention, while non-pharmacological interventions, which have been shown to be effective, have tended to have limited implementation (3).

In a highly medicalised society with an increasing demand for healthcare, primary care services are particularly overburdened, limiting their full potential for health promotion or even impairing users' health in some cases. The efficiency of the system is called into question if users are forced to go to hospital emergency departments because they cannot have a timely appointment with their primary care physician (4). However, the problem goes beyond inefficiency: overburdened health services have a direct impact on health equity, widening the gap between the most and least well-off members of society. It has been shown that people with lower educational attainment endure longer waiting times and greater negative impact of shorter primary care visits (4, 5). Barriers to accessing health services affect the population unequally, with the poorest and most vulnerable groups being the most likely to experience these barriers (6).

Given the current context, the CAIR project (Co-creating Action to Improve Rationality in the health system) aims to stimulate changes in health system functioning and increase health equity by the co-creation of community-based initiatives to improve health and wellbeing in the Municipal District 2 of Elche, Spain. Co-creation, in this regard, is defined as a collaborative process that brings together different actors to design, implement and evaluate interventions aimed at improving population health. This approach includes the participation of diverse sectors, such as policy makers, health professionals, community organizations and citizens, working together to identify problems and develop and implement solutions in a participatory procedure. The process will incorporate an asset-based approach to health (7) by identifying and mobilizing community's existing assets. This approach can promote a sense of ownership and self-determination among community members, thereby strengthening their capacity to drive improvements in wellbeing and community health (8, 9).

Given its focus on medicalisation, the CAIR project targets three thematic domains in which overreliance on biomedical solutions and patterns of inappropriate or unnecessary treatment are especially evident. First, psychological wellbeing, with the aim of reducing unnecessary consumption of anxiolytics and antidepressants by strengthening community-level strategies that promote mental health and resilience. Second, cardiovascular health, where fostering preventive, community-based approaches can reduce dependence on medication to manage cardiovascular risk factors. Third, antimicrobial resistance, by promoting the rational use of antibiotics and supporting behaviors that help limit the emergence and spread of resistance. Together, these domains reflect key areas in which co-created, community-based initiatives may help counteract medicalisation while improving health, promoting equity, and reducing avoidable pressure on primary care services.

This protocol describes the population surveys that constitute a core component of the CAIR project. The surveys will be conducted twice during the project period—before and after the co-creation process—to capture changes over time in key indicators at the municipal district level. The surveys will generate quantitative evidence on the potential effects of moving from a predominantly biomedical model to one that embeds community participation and collective action in efforts to improve health.

Therefore, the aim of this study is to analyse changes in health-related quality of life, critical health literacy, social and community capital, and knowledge, attitudes, and practices regarding personal antibiotic use—and their implications for health equity—through population surveys conducted before and after the co-creation process in the Municipal District 2 of Elche, Spain.

## 2 Methods and analysis

### 2.1 Study design

The CAIR study applies a community-based participatory approach to engage citizens in co-creating initiatives to improve health and wellbeing. To evaluate their effect in the Municipal District, we will conduct representative population-based surveys before and after the three-year co-creation period (2026–2028). These surveys will measure health-related quality of life, critical health literacy, social and community capital, and knowledge, attitudes, and practices related to personal antibiotic use, alongside key sociodemographic characteristics to enable an equity-focused analysis. Baseline results will inform the co-creation process by identifying community needs, health status and health inequities, ensuring that initiatives are designed to address them effectively and fairly. Changes over time will be assessed by comparing indicators between surveys before and after the co-created intervention process, also estimating absolute and relative differences across population groups.

The broader CAIR project is registered on ClinicalTrials.gov (Identifier: NCT07237971). CAIR began on 1 October 2024 and will run for 5 years. Recruitment for the baseline survey started on 22 October 2025 and the post-intervention survey will be conducted in the final year, in 2029.

### 2.2 Study setting

**Figure 1:**
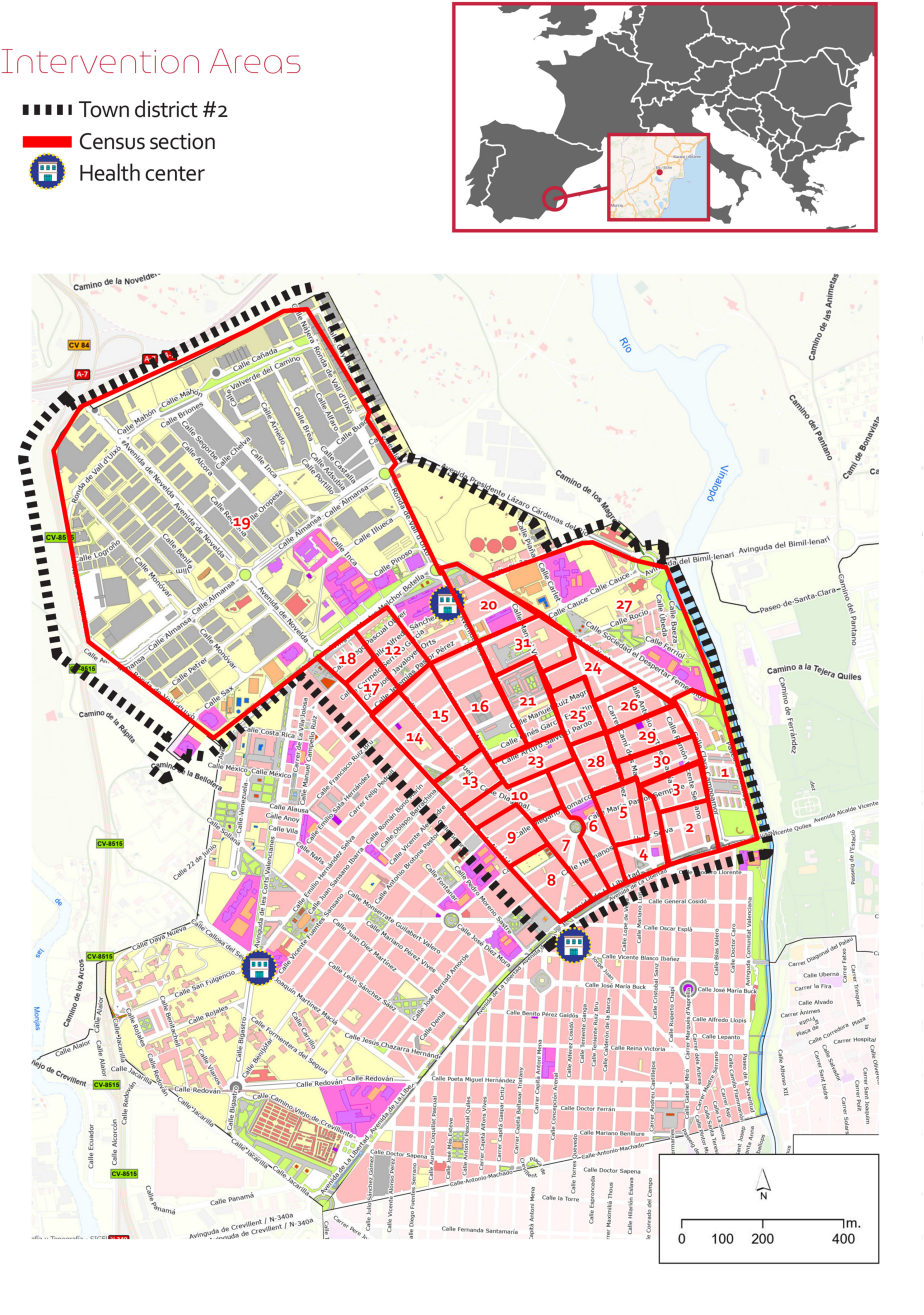
Geographic distribution of census sections in the study area: Municipal District 2, Elche, Spain. According to the Elche City Council, this area is the Municipal District 2. In the National Institute of Statistics (INE) statistical classification, it is District 3.

We will conduct a representative population-based survey among adult residents (≥18 years) in Municipal District 2 of Elche, Spain (Figure 1). This district includes the neighborhood of Carrús where resides 17.2% of the population of the city of Elche, with 36,065 residents in 2024. It is a diverse area, with 25.6% foreign-born residents, mainly from Morocco (6.2%), Colombia (5.3%), and Romania (3.1%) (10), as well as approximately 250 Roma families (11). The area shows levels of social vulnerability, with a high concentration of migrants and lower income levels. The Roma community faces structural discrimination and barriers to accessing education and employment, which negatively affect their wellbeing and opportunities (11).

We will work closely with the basic health councils of the two health centers that have a population residing in District 2 of Elche (Carrus Health Center and San Fermín Health Center). Basic Health Councils are established structures that promote citizen participation in health, bringing together representatives from the primary care team, local government, public health services, neighborhood associations, community organizations, and other relevant actors (12). Their role will be essential for mobilizing the local population and facilitating participation in the survey.

### 2.3 Study population

We will recruit 600 participants using a stratified random sampling based on the 2021 census sections. The sampling will be stratified by census section, sex, age group to ensure its representativeness using population quotas from census data provided by Spain's National Statistics Institute (INE, Supplementary Table S1)(10). Furthermore we will apply an overall migrant quota of 25.6%. By using a stratified sampling, we ensure a representative sample of the neighbourhood's population, also in terms of representativeness of men and women across age groups for each of the different census sections that compound the District 2 of Elche. Furthermore, given that representativeness is a challenge for any population survey, targeted outreach through social media, local traditional media and community-based engagement activities will be employed to increase community awareness and improve response rates.

#### 2.3.1 Inclusion and exclusion criteria

- Inclusion criteria:
  (a)People over 18 years of age who reside in the Municipal District 2 of Elche (Spain) (after providing informed consent to participate in the survey).
- Exclusion criteria:
  (a)People who cannot understand the questions or give informed consent due to language or cultural barriers, even after attempting to establish effective communication with the assistance of a cultural mediator.
  (b)People with physical or intellectual disabilities that prevent them from understanding the questions or signing the informed consent, despite reasonable efforts to facilitate communication.

### 2.4 Sample size estimation and selection criteria

The sample size has been calculated assuming that the prevalence of the variables of interest (e.g. proportion of individuals with poor health-related quality of life, proportion of individuals with limited critical health literacy etc.) does not exceed 30%, with an absolute precision of ± 5% and a confidence level of 95%. A design effect of 1.5 has been applied, following the recommendations of the World Health Organization's (WHO) STEPS guidelines (13) for complex designs. In addition, a rejection rate of up to 20% has been considered, implying the need to recruit at least 590 participants. The proposed inclusion of 600 participants also ensures the estimation of indicators for specific subgroups (such as by sex) with an absolute precision of close to ± 5%.

A before-and-after analysis will be conducted with 600 independent participants at each time point, selected using comparable samples and applying the same inclusion criteria and sampling design. This sample size is sufficient to detect significant differences in both our main outcome (i.e., health-related quality of life) and secondary variables. In the case of health-related quality of life, it allows us to identify absolute changes of around 10%, with a confidence level of 95% and a statistical power close to 80%. The exact power will depend on the initial prevalence of the indicator and the magnitude of the observed effect. Although the analysis of social inequalities may have lower power due to stratification by subgroups, we estimate that the sample size will be sufficient to identify relevant trends and significant differences in social inequalities in the studied sample.

### 2.5 Documentation and classification of co-creation cycles

The development of new initiatives is expected to follow a cyclical process, in which community stakeholders collaboratively identify priorities, co-design and implement actions, and iteratively evaluate and adapt them, informed by the RE-AIM framework (Reach, Effectiveness, Adoption, Implementation, and Maintenance). To document each cycle, a standardized matrix will be used to record key characteristics, including a brief description of the activities developed, target population, setting, intensity and duration, level of community involvement, and evaluation results (quantitative and qualitative). In line with the project's Data Management Plan, structured outputs from each cycle—including short reports and infographics summarizing key outcomes, agreements, and process evaluation findings—will be made available through the project's open-access repository (i.e., Zenodo). This approach will enable cycles to be grouped into analytically meaningful categories while preserving relevant contextual detail.

### 2.6 Procedure

For each sampling route, a random point within the census section will be selected to determine the starting location for data collection. From there, a random sample of households will be chosen along the route, and finally, one individual per household will be recruited according to the quota strategy (Supplementary Table S1).

A team of four trained interviewers will conduct face-to-face the data collection in the sampled households or, when appropriate, in workplaces, as explained below. Using tablet devices, they will first provide participants a detailed explanation of the purpose of the study, the confidentiality of their responses, and their right to withdraw at any time without consequences. Written informed consent will then be obtained from all participants.

Surveys may be conducted in workplaces or other communal spaces in the community when an imbalance in the sample—particularly an under-representation of the working-age population—has been identified following household-based recruitment efforts. In this case, participation will be offered only to individuals whose residence in the study area has been previously verified.

In addition, we will have the support of the intercultural mediators, previously trained in public health by the Elche Public Health Center to address possible linguistic or cultural barriers to participation for the Arabic-speaking population. If people with communication difficulties are identified, a specific day will be organized to deal exclusively with these cases, with the assistance of one or more intercultural mediators who will assist the team of interviewers in person or by phone. These mediators will receive specific training in the administration of the questionnaire, the use of the electronic devices and compliance with the study's ethical protocol. This approach ensures the inclusion and participation of all segments of the population, including hard-to-reach persons.

### 2.7 Survey tool and variables

The survey is structured in five main sections (sociodemographic, health-related quality of life, critical health literacy, social connectedness, community capital, and knowledge, attitudes and practices on personal antibiotic use). The first section, which collects sociodemographic characteristics, will always be administered at the beginning of the survey. Participants will self-fill these questions on the interviewer's device, unless they request assistance, in which case the interviewer will read them out. This sociodemographic information will be used to analyse health inequalities within the study area, disaggregating the results by relevant social groups (by age, country of birth, race/ethnicity, sex, gender, etc.) and calculating both absolute and relative inequalities. The remaining four sections will be presented in a random order for each participant, using an algorithm embedded in the electronic form. This randomization of the remaining four sections (health-related quality of life, critical health literacy, social connectedness, community capital, and knowledge, attitudes and practices regarding personal antibiotic use) will allow the random and equitable distribution of potential biases in the understanding and answering of the questions, especially those at the end of the survey. The complete survey tool, variables, and response options can be found at the Supplementary Information of this protocol (Supplementary Supplementary Tables S2–S7).

The main outcome of the surveys is the health-related quality of life (HRQoL) measured using the VR-12 questionnaire. This tool has been internationally validated to measure HRQoL e (14). The VR-12 questionnaire contains 12 items that assess 8 aspects of health-related quality of life: physical functioning, physical role, bodily pain, general health, vitality, emotional role, social functioning and mental health. The VR-12 questionnaire measures these aspects in scales to produce a summary score in two dimensions: a physical dimension, represented by the Physical Component Summary, and a mental dimension, represented by the Mental Component Summary.

In addition, as secondary variables, a series of indicators will be measured with a descriptive and analytical purposes to also guide the co-creation process and a complementary role in the impact assessment as intermediate outcomes.

*Critical Health Literacy (CHL)*. We will measure CHL (15) using the All Aspects of Health Literacy Scale (AAHLS) (16) after analyzing the psychometric properties of a new version adapted to Spain. CHL comprehends the capacity to critically analyze health determinants, reflect on socioeconomic and cultural contexts, which promotes advanced personal skills, interactive engagement with health systems, informed decision-making, empowerment, and the effectiveness of health promotion interventions (17, 18).

*Community capital*. We have developed a tool to measure the perception and use of community resources, defined as “any factor (or resource) that enhances the capacity of individuals, groups, communities, populations, social systems and institutions to maintain and sustain health and wellbeing and helps them to reduce health inequalities” (8). The tool classifies resources in seven categories: 1. Natural, 2. Cultural, 3. Human, 4. Social, 5. Built, 6. Financial and 7. Political, in line with the community assets literature (19, 20). Participants will be asked to describe the resources in their environment (neighborhood where they live, and other places in the municipality that they frequent) in each category, and the interviewer will record yes or no accordingly (Supplementary Table S5). We will then ask participants about the frequency of use and when appropriate, the perceived barriers that prevent them from making more use of these community assets. The list of potential barriers was prepared based on literature review (21, 22). The instrument was pre-tested to obtain feedback on the clarity and relevance of the questions in order to make any necessary adjustments before its final implementation.

*Social capital and community cohesion*. Here, we draw on items from a previously validated scale on bridging social capital used in the United States with Latino immigrant populations (23), complemented by selected questions adapted from the Canadian Community Health Survey (24), related to a sense of belonging to one's community and trust. The resulting instrument was tested through a pilot study, and its psychometric properties will be evaluated to ensure its validity and suitability for our local context.

*Knowledge, attitudes and practices (KAP) on personal antibiotic use*. We will measure KAP on personal antibiotic use using an instrument validated in Spain (25). This will enable us to obtain an analysis of common antibiotic-related behaviors by analyzing the following three dimensions: knowledge (what respondents know about antibiotics), attitude (what respondents think about antibiotics) and practice (what they do regarding antibiotics).

For the new validated versions of AAHLS, social capital and community cohesion, and community capital scales, a pilot test will be conducted on 20 participants to evaluate the clarity of the adapted version of the questionnaire before conducting the survey. The initial versions were subsequently reviewed by an external panel of experts in epidemiology, psychology and both English and Spanish languages to refine the translation and minimize potential misunderstandings among respondents.

To analyse their psychometric properties, we will use Cronbach's alpha, Confirmatory Factor Analysis (CFA) and bivariate analyses. Cronbach's alpha will assess the internal consistency of each scale. CFAs will be conducted to evaluate whether the data fitted the theoretical factor structure proposed by the original scales of AAHLS and social capital and community cohesion as well as the proposed structure of the community capital tool. Model fit will be evaluated using standard indices. The Comparative Fit Index (CFI) and Tucker–Lewis Index (TLI) will assess the relative improvement of the tested model over a null model, with values approaching 0.90 indicating acceptable fit. The Root Mean Square Error of Approximation (RMSEA) will evaluate absolute model fit, with lower values indicating better approximation of the data. Finally, bivariate analyses will complement the CFA by examining expected associations between scale scores and sociodemographic variables.

### 2.8 Data analysis

As main outcome, the mean and the standard deviation of the health-related quality of life will be calculated and stratified by sex, age group and other sociodemographic variables, thus allowing for an equity analysis. In addition, we will perform multivariate models to analyse the association of sociodemographic characteristics on health-related quality of life and secondary variables (critical health literacy, social capital, community capital, and knowledge, attitudes and practices on personal antibiotic use). These results will be also crucial to enrich the discussions in the co-creation process, facilitating an equitable and contextualized approach to the identified health issues. A before-and-after analysis will also be carried out using the population-based survey data to assess changes across time points (pre- and post-intervention) in the population in both main and secondary variables, providing evidence on the evolution of health-related quality of life and other relevant social determinants of health. Specifically, firstly, the normal distribution of physical and mental HRQoL will be tested by conducting Shapiro-Wilk tests and plotting histograms. In case of observing a non-normal distribution, these variables will be dichotomised using the median value of the whole sample (pre- and post-intervention). Therefore, given the distribution of health-related quality of life, multivariate linear regression models or Poisson regression models with robust standard errors will be used to estimate absolute and relative changes across time points. Additional models will be run, including interaction terms of time with sex, age group and other sociodemographic variables to assess the changes in social inequalities in HRQoL. Contextual variables will be included, such as economic development, unemployment rate and poverty rate at the regional level of the province of Alicante before and after the intervention, retrieved from the National Institute of Statistics (INE) and the statistics portal of the Valencian Community in Spain. Statistical analysis will be adjusted for sex, age group and other sociodemographic variables such as education, ethnicity, migration status, household income, employment status and marital status, among others. Multiple imputation will be used for missing data on HRQoL and secondary variables. For the remaining covariates, an additional category of missing values will be created to include these individuals in the statistical analyses.

For statistical analysis, we will use the latest version of Stata/SE (StataCorp, Texas, USA), and R: A language and environment for statistical computing (R Foundation for Statistical Computing, Vienna, Austria). The *p*-value obtained in the hypothesis tests performed will be described. Although we will use a *p*-value < 0.05 as a reference to identify statistically significant differences, this value should be interpreted with caution when subgroup stratified analyses with smaller sample sizes are conducted. The possibility of a type II error (false acceptance of the null hypothesis) will be considered, allowing for a more nuanced and contextualized interpretation of the results, thus avoiding the premature exclusion of potentially relevant trends in under-represented subgroups. No survey weights will be used in statistical analyses.

## 3 Discussion

In a context of increasing medicalisation and an overburdened health system, non-pharmacological interventions based on community-based approaches are often relegated to the background, despite their proven efficacy at the population level (26). Although many health professionals recognize the potential and importance of these interventions for patients, they also point out that lack of time and accessible resources limit their implementation in daily practice (27, 28). Within this framework, the CAIR project will provide evidence to the growing recognition that improving health equity requires moving beyond individual-level interventions toward approaches that engage communities in defining and addressing their own priorities. The population-based surveys described here will allow us to assess the effect of the co-created intervention on health-related quality of life, critical health literacy, social and community capital, and knowledge, attitudes and practices on personal antibiotic use as well as their social inequalities across sociodemographic information.

HRQoL was selected as our primary outcome because it is increasingly recognized in epidemiological research as a robust indicator of morbidity and mortality, capturing not only physical health but also mental and social wellbeing (29, 30). Examining variations in HRQoL across sociodemographic groups will enable an equity-focused analysis and provide critical insights into the health of the local population. This information will be essential for assessing the effect of the co-created process for advancing toward health equity. The multidimensional structure of the VR-12 questionnaire further allows us to identify health-related inequalities across demographic characteristics and to evaluate changes in both physical and mental health domains after the intervention (31, 32).

We will also incorporate CHL into our analysis. According to WHO, strategies that focus on the design and implementation of interventions that address health inequalities should include an assessment of the health literacy of communities. Different domains of health literacy include basic functional health literacy (the ability to access, understand, appraise and apply health information), communicative health literacy and critical health literacy (15). In this project we are particularly concerned with CHL because it involves the critical appraisal of information, understanding the social determinants of health and is linked to collective action (18). Furthermore, improved critical health literacy is widely assumed to lead to a more effective and efficient use of services (17).

Additionally, we have designed a tool to measure community capital, considered a central dimension for understanding how local contexts shape opportunities for health and wellbeing. Building on the principles of salutogenesis and the assets-based community development literature (9, 19), our tool seeks to gather information that promotes the recognition and mobilization of the resources, relationships, and strengths that already exist within communities. However, previous research has shown that merely identifying or mapping community assets does not necessarily translate into meaningful change (8). Resources often remain underused when individuals face social, cultural, economic, or structural barriers to accessing them. These barriers are themselves socially patterned and can exacerbate inequities if not addressed. One of the innovative contributions of this study is the development of a tool that not only provides a quantitative picture of the community capital individuals perceive, but also captures the barriers they experience in practice and how these vary across population groups. We hope that this nuanced understanding will be particularly valuable for the cocreation of community initiatives within the CAIR project, enabling strategies that both strengthen community capital and actively tackle the obstacles that limit its equitable use.

Although social capital is included in the community capital scale described above, we added a more explicit focus on this dimension given its relevance as a key determinant of physical and mental health (33–35) and its influence on participation in community processes (36). Social capital can be conceptualized at both individual and community level. While cognitive social capital refers to people's perceptions about the level of interpersonal trust, sharing and reciprocity in their community, on the other hand, structural social conectiveness includes the density of one's social networks, and patterns of civic engagement (35). Furthermore, we can think of “bonding” capital when members of your network provide support in times of strife (e.g., cover costs in moments of need) or “bridging” capital when people in your network can help you improve your situation (e.g., relatives able to help you achieve goals). The tool proposed in this study includes items capturing assistance-related trust (e.g., receiving financial help in times of need), contact with similar or different people as an indicator of bridging social capital, and sub-section on community belonging and cohesion. Improving social capital and its different dimensions is a likely benefit from our project's research activity itself, and is a key determinant of physical and mental health (33, 34).

Finally, we included a questionnaire to measure KAP regarding antibiotics at the population level. This is directly relevant to the rational use of antibiotics and, consequently, to reducing the risk of AMR, which is recognized as one of the top ten threats to global public health (37). Although a comprehensive approach to AMR must also consider prescribing practices among professionals and antibiotic use, the overuse and misuse of antibiotics in the community remains a major driver of AMR, which requires urgent, coordinated action (38). Misconceptions about antibiotics are widespread, such as believing they are effective against viral infections or discontinuing treatment once symptoms improve (39). Existing communication and engagement initiatives often miss the most vulnerable and those most at risk of suffering the negative consequences of AMR (40). Therefore, reducing popular demand for antimicrobials is a key objective of any effective AMR strategy, and the KAP data collected here will help inform targeted, equity-oriented interventions within the CAIR project.

It is essential to adopt more holistic approaches that combine biomedical interventions with non-pharmacological strategies, promoting active community participation and addressing both the underlying causes of health problems and the social inequalities that shape them. The implications of the study are not only on analyzing the potential effects of co-created actions on health and health equity, but also on generating evidence to decision-makers that community-centered, intersectoral approaches may strengthen health system performance and contribute meaningfully to better outcomes at a population level.

The use of repeated population surveys is a strength of this study, as it enables the assessment of change over time through before-and-after comparisons of carefully selected indicators that are directly relevant to co-creation processes. However, an important limitation of the overall design is the absence of a control area where no co-creation activities are implemented. As this is not a controlled trial, we cannot fully disentangle the effects of the interventions from broader contextual changes occurring over the same period. Therefore, the results obtained from the before-and-after analysis should be interpreted with caution. Various external and contextual factors, such as other policy measures implemented for the general population, economic and social changes, or changes in access to healthcare, may also influence the evolution of our primary health outcome despite controlling for macro-level variables. Moreover, different, independent participants will be recruited at baseline and after the intervention, which also limits the effects attributable to the co-creation intervention process. In this regard, our analysis should be interpreted as time-trend changes in the Municipal District 2 of Elche where targeted cocreation is taking place rather than changes among individuals taking part in the cocreation intervention.

For the surveys themselves, we also recognize potential challenges related to the underrepresentation of migrant populations, particularly those in more vulnerable situations. In this sense, such population groups—particularly migrants and racialised populations—are more likely to report poorer health outcomes and if they are underrepresented in the sample, the resulting estimates may be biased upwards, overstating the overall health status of the population. To mitigate this, the role of cultural mediators will be crucial to ensure culturally competent engagement and reduce barriers to facilitate the participation of such groups and Arabic-speaking individuals.

## 4 Conclusion

This protocol outlines the design of the population-based survey component of the CAIR study. The survey will provide essential baseline and post-intervention data to assess changes in key indicators of health-related quality of life, critical health literacy, social and community capital, and knowledge, attitudes, and practices on personal antibiotic use. Integrated within a participatory process of co-creation, the surveys will enable a before-and-after assessment of community-level changes and provide valuable insights into the measurement of health equity in complex, real-world settings.
