# Supplementary material for: Assessing the impact of co-created community initiatives on health and equity: a protocol for population-based surveys within the CAIR project in Elche, Spain
Source: Front Public Health. 2026 Jun 3;14:1766000. doi: 10.3389/fpubh.2026.1766000 (PMC13274431; doi:10.3389/fpubh.2026.1766000)
Supplement: Supplementary file 1 [file Data_Sheet_1.docx]

Supplementary Material for:

Assessing the impact of co-created community initiatives on health and equity: a protocol for population-based surveys within the CAIR project in Elche, Spain

**This file includes:**

[**Table S1.** Age, sex and migrant population quotas for population survey, estimated with census data in the Municipal District 2 of Elche, Spain 2](#_heading=h.w9bhumf58vtg)

[**Table S2.** Questionnaire module on sociodemographic characteristics. 3](#_heading=h.t13twnb6q6t5)

[**Table S3.** Questionnaire module on health-related quality of life: VR12 (1) 8](#_heading=h.n56d1ga9tbv)

[**Table S4.** Questionnaire module to assess critical health literacy: All aspects of Health Literacy Scale (AAHLS) (2) 9](#_heading=h.yd3whygwgauc)

[**Table S5.** Questionnaire module on community capital and barriers to use 10](#_heading=h.j25m6ayqtq33)

[**Table S6.** Questionnaire module on social capital and community cohesion (3,4) 15](#_heading=h.gu77ewe05srl)

[**Table S7:** Questionnaire module on knowledge, attitudes and practices on personal antibiotics. (5) 17](#_heading=h.nsgm1n12bo1a)

## **Table S1.** Age, sex and migrant population quotas for population survey, estimated with census data in the Municipal District 2 of Elche, Spain

(native = 468; migrant = 132; total = 600)

|  | **Men (years)** | | | | **Women (years)** | | | |  |
| --- | --- | --- | --- | --- | --- | --- | --- | --- | --- |
| **Census section** | **18 – 34** | **35 – 49** | **50 – 64** | **≥65** | **18 – 34** | **35 – 49** | **50 – 64** | **≥65** | **Total** |
| 3001 | 2 | 2 | 2 | 1 | 2 | 1 | 2 | 2 | **14** |
| 3002 | 3 | 3 | 3 | 2 | 2 | 2 | 3 | 3 | **21** |
| 3003 | 2 | 3 | 2 | 3 | 2 | 2 | 3 | 3 | **20** |
| 3004 | 2 | 2 | 2 | 2 | 2 | 2 | 2 | 3 | **17** |
| 3005 | 3 | 2 | 3 | 2 | 3 | 2 | 3 | 3 | **21** |
| 3006 | 3 | 3 | 2 | 2 | 3 | 3 | 2 | 2 | **20** |
| 3007 | 3 | 3 | 3 | 2 | 3 | 4 | 4 | 3 | **25** |
| 3008 | 3 | 3 | 2 | 2 | 2 | 2 | 3 | 3 | **20** |
| 3009 | 3 | 3 | 2 | 2 | 3 | 2 | 3 | 2 | **20** |
| 3010 | 4 | 5 | 3 | 2 | 4 | 4 | 3 | 3 | **28** |
| 3012 | 3 | 4 | 3 | 2 | 3 | 4 | 4 | 3 | **26** |
| 3013 | 2 | 3 | 3 | 1 | 2 | 2 | 2 | 2 | **17** |
| 3014 | 3 | 4 | 3 | 2 | 2 | 4 | 2 | 2 | **22** |
| 3015 | 3 | 4 | 4 | 2 | 3 | 4 | 3 | 3 | **26** |
| 3016 | 3 | 4 | 4 | 2 | 3 | 4 | 3 | 3 | **26** |
| 3017 | 2 | 2 | 2 | 2 | 2 | 2 | 2 | 2 | **16** |
| 3018 | 2 | 2 | 2 | 2 | 2 | 3 | 1 | 2 | **16** |
| 3019 | 2 | 2 | 2 | 2 | 1 | 2 | 2 | 2 | **15** |
| 3020 | 2 | 2 | 3 | 2 | 2 | 2 | 5 | 3 | **21** |
| 3021 | 4 | 3 | 4 | 3 | 3 | 3 | 3 | 4 | **27** |
| 3023 | 3 | 3 | 2 | 1 | 3 | 3 | 2 | 2 | **19** |
| 3024 | 3 | 3 | 3 | 2 | 3 | 3 | 3 | 3 | **23** |
| 3025 | 3 | 3 | 2 | 1 | 2 | 3 | 2 | 2 | **18** |
| 3026 | 2 | 2 | 3 | 2 | 2 | 2 | 2 | 3 | **18** |
| 3027 | 3 | 4 | 4 | 2 | 3 | 3 | 2 | 2 | **23** |
| 3028 | 3 | 3 | 2 | 1 | 2 | 2 | 1 | 2 | **16** |
| 3029 | 4 | 4 | 3 | 3 | 3 | 4 | 2 | 3 | **26** |
| 3030 | 3 | 3 | 3 | 2 | 2 | 2 | 2 | 3 | **20** |
| 3031 | 3 | 2 | 3 | 2 | 2 | 2 | 3 | 2 | **19** |
| **Total** | **81** | **86** | **79** | **56** | **71** | **78** | **74** | **75** | **600** |

## **Table S2.** Questionnaire module on sociodemographic characteristics.

| What is your Health Centre? | - Carrús - San Fermín - Other (specify) - Don’t know - No answer provided |
| --- | --- |
| What year were you born? |  |
| What is your country of birth? | - Spain - Other (specify) - Don’t know - No answer provided |
| What province were you born in? *[Question only if participant selects Spain]* |  |
| In what year did you arrive in Spain? *[Question only if participant does not select Spain]* | - Year of arrival in Spain: - Don’t know - No answer provided |
| In what year did you arrive in Elche? *[Question only if participant does not select Alicante province]* | - Year of arrival in Spain: - Don’t know - No answer provided |
| What is your nationality or nationalities? You may select more than one option if you have dual citizenship *[Question only if participant does not select Spain]* | - Spain - Other (specify) - Don’t know - No answer provided |
| Which of the following documents do you currently have? | - Permanent residence permit - Temporary residence permit - Residence application submitted - Residence application not yet submitted - Refugee status - Asylum seeker - No documents listed above - Other (specify) - Don't know - No answer provided |
| What is your father/mother’s country of birth? | - Spain - Other (specify) - Don't know - No answer provided |
| In which province was he/she born in? *[Question only if participant selects Spain in previous question]* |  |
| What is the country of birth of your other parent? | - Spain - Other (specify) - Don’t know - No answer provided |
| In which province was he/she born in? *[Question only if participant selects Spain in the previous]* |  |
| Based on your family history, ancestry, sense of belonging, and culture, which of the following best describe you. You may select multiple answers. | - White European - White Latin American - Black/Afro/Afro-descendant/Afro-Spanish/Black African - Romani/Roma - Arab - Amazigh/North African non-Arab - Native American, Indo-American/Indigenous/native of Abya Yala - East or Southeast Asian - South-Central Asian - West Asian/Turkish - Mixed/Mestizo/Various ancestries - Other (specify) - I don't know - I prefer not to answer this question - I don't understand this question |
| Could you tell us what sex you were assigned at birth? | - Female - Male - Intersex/Intersexual |
| Which gender do you identify with? | - Woman - Man - Non-binary - None of these options represent my gender - I prefer to describe myself as:(specify) |
| Which of the following best describes you? | - Heterosexual - Homosexual/Gay/Lesbian - Bisexual - Asexual - Don't know - No answer provided - I prefer to describe myself as:(specify) |
| What is your marital status? | - Single - Married - Common-law partner - In a relationship (cohabiting without being married) - Separated or divorced - Widowed - Other (specify) - Don't know - No answer provided |
| Have you attended school or pursued any type of study? If so, what is the highest level of education you have attained? | - I have not attended school or pursued any studies, and I cannot read or write - I have not attended school or pursued any studies, but I can read and write - Yes, I have attended school and completed (or not completed) primary studies. Examples: primary school, school graduation certificate, EGB (General Basic Education), school certificate, 1st and 2nd year of ESO - first cycle - up to age 14 - Yes, I have attended school and completed (or not completed) secondary studies. Examples: ESO, Bachillerato (Spanish upper secondary education), COU (University Orientation Course), Vocational Training, or studies that require a primary education certificate for admission - Yes, I have attended school and completed (or not completed) university studies. Examples: Diploma, Bachelor's degree, Engineering degree, Master's degree, Doctorate Don't know - No answer provided |
| Number of people residing in your household (including yourself) |  |
| How many of these people are minors? If there are no minors in the household, enter 0. |  |
| And how many are under 15 years of age? If there are no persons under 15 years of age in the household, enter 0. |  |
| Who is responsible for the care of these persons under the age of 15? | - Mainly me, the person answering the questionnaire - Mainly someone else - Me and someone else sharing 50% or almost 50% - Don't know - No answer provided |
| Do any persons live in your household who, due to a limitation or disability, are unable to care for themselves and require the care of another person? Do not include infants or children without disabilities | - Yes - No - Don't know - No answer provided |
| Who is primarily responsible for their care? | - Mainly me, the person answering the questionnaire - Mainly someone else - Me and someone else sharing 50% or almost 50% - Don't know - No answer provided |
| Do you have an officially recognised disability? | - Yes - No - Don't know - No answer provided |
| What is your officially recognised degree of disability? | - Less than 33% - From 33% to 64% - 65% or more - Don't know - No answer provided |
| In your household, who is primarily responsible for household chores such as washing, cooking, ironing, etc? | - Just me, the person answering the questionnaire - Me sharing it with another person 50% or almost 50% - Another person in the household mainly - A person who does NOT live in the household, paid for doing so - Another situation - Don't know - No answer provided |
| What is your current employment status? | - I receive remuneration for work (working) - I receive remuneration for work and I also study (working and studying) - I am exclusively engaged in housework - I receive benefits (widow’s pension, incapacity for work, etc.) - I am exclusively a student - I do not work but I am looking for work - I am retired and receive a retirement pension - Other (specify) - Don't know - No answer provided |
| How long have you been in this situation? *[Question only if the person is not working and looking for a job]* |  |
| What is your main occupation? | - Directors and managers - Scientific and intellectual technicians and professionals - Support technicians and professionals - Accounting, administrative and other office employees - Catering, personal and health service workers, security guards and salespersons - Skilled workers in agriculture, livestock, forestry and fishing - Craftsmen and skilled workers in manufacturing and construction (except plant and machine operators) - Plant and machine operators and assemblers - Elementary occupations (unskilled workers in services, labourers in agriculture, fishing, construction and industry, etc) - Military occupations - Don't know - No answer provided |
| What type of contract or employment relationship do you have? | - Employee (salaried, commission, daily wage, etc) with contract - Employee without contract - Entrepreneur or professional with employees - Entrepreneur or professional without employees - Family helper (without regulated remuneration in the family business or company) - Member of a cooperative - Another situation (specify) - Don't know - No answer provided |
| What is the duration of your contract? *[Question only if the person is an employee with a contract]* | - Under 6 months - From 6 months to less than 1 year - From 1 year to less than 2 years - 2 years or more - Permanent contract - Permanent seasonal contract - Civil servant - Another situation (specify) - Don't know - No answer provided |
| Is your main job full-time or part-time? | - Full-time - Part-time - Don't know - No answer provided |
| Was it you who decided to work full-time or part-time? | - Yes - No - Don't know - No answer provided |
| Could you indicate the approximate net monthly income for your entire household? | - Less than €1,000 - Between €1,000 and €1,800 - Between €1,800 and €3,000 - More than €3,000 - Don't know - No answer provided |

## **Table S3.** Questionnaire module on health-related quality of life: VR12 (1)

| In general, would you say your health is: | - *Excellent* - *Very good* - *Good* - *Fair* - *Poor* |
| --- | --- |
| The following statements are about activities you might do during a typical day. Does your health now limit you in these activities? If so, how much? | |
| - Moderate activities, such as moving a table, pushing a vacuum cleaner, or playing bowling or golf... | *Answer options for each item:*   - *Yes, limited a lot* - *Yes, limited a little* - *No, not limited at all* |
| - Climbing several flights of stairs… |  |
| During the past 4 weeks, have you had any of the following problems with your work or other regular daily activities as a result of your physical health? | |
| - Accomplished less than you would like | *Answer options for each item:*   - *No, none of the time* - *Yes, a little of the time* - *Yes, some of the time* - *Yes, most of the time* - *Yes, all of the time* |
| - Were limited in the kind of work or other activities |  |
| During the past 4 weeks, have you had any of the following problems with your work or other regular daily activities as a result of any emotional problems (such as feeling depressed or anxious)? | |
| - Accomplished less than you would like | *Answer options for each item:*   - *No, none of the time* - *Yes, a little of the time* - *Yes, some of the time* - *Yes, most of the time* - *Yes, all of the time* |
| - Didn't do work or other activities as carefully as usual |  |
| During the past 4 weeks, how much did pain interfere with your normal work (including both work outside the home and housework)? | - *Not al all* - *A little bit* - *Moderately* - *Quite a bit* - *Extremely* |
| These questions are about how you feel and how things have been with you during the past 4 weeks. How much of the time during the past 4 weeks… | |
| - have you felt calm and peaceful? | *Answer options for each item:*   - *All of the time* - *Most of the time* - *A good bit of the time* - *Some of the time* - *A little of the time* - *None of the time* |
| - did you have a lot of energy? |  |
| - have you felt downhearted and blue? |  |
| Now, we’d like to ask you some questions about how your health may have changed... | |
| - compared to one year ago, how would you rate your physical health in general now? | *Answer options for each item:*   - *Much better* - *Slightly better* - *About the same* - *Some of the time* - *Slightly worse* - *Much worse* |
| - compared to one year ago, how would you rate your emotional problems (such as feeling anxious, depressed or irritable) now? |  |

## **Table S4.** Questionnaire module to assess critical health literacy: All aspects of Health Literacy Scale (AAHLS) (2)

| - How often do you need someone to help you when you are given information to read by your doctor, nurse, pharmacist, psychologist, physiotherapist, podiatrist, etc.? - When you need help, can you easily get hold of someone to assist you? - Do you need help to fill in official documents? - When you talk to a doctor or nurse, do you give them all the information they need to help you? - When you talk to a doctor or nurse, do you ask the questions you need to ask? - When you talk to a doctor or nurse, do you make sure they explain anything that you do not understand? - Are you someone who likes to find out lots of different information about your health? - How often do you think carefully about whether health information makes sense in your situation? - How often do you try to work out whether information about your health can be trusted? | *Answer options for each item:*   - Often - Sometimes - Rarely |
| --- | --- |
| - Are you the sort of person who might question your doctor or nurse’s advice based on your own research? - Do you think that there plenty of ways to have a say in what the government does about health? | - Yes, definitely - Maybe-sometimes - Not really |
| Within the last 12 months have you taken action to do something about a health issue that affects your family or community? | - Yes - No |
| What do you think matters most for everyone’s health? (tick one answer only) | - Information and encouragement to lead healthy lifestyles. - Good housing, education, decent jobs and good local facilities |

## **Table S5.** Questionnaire module on community capital and barriers to use

*[Introductory text to be read by interviewer]* In this section of the survey, we will talk about the resources that exist in a community that help maintain and improve the wellbeing and health of individuals and the community.

| ***[Natural capital]***  Let’s start by talking about green spaces, including open areas with vegetation such as parks, orchards, gardens, forests, lakes, rivers, and natural landscapes, all of which provide benefits for our physical and mental health | |
| --- | --- |
| What natural areas or green spaces do you know in your neighbourhood? | *Answer options for each group:*   - *Yes* - *No* - *No answer provided*   *If the participant mentions any, the interviewer marks ‘Yes’* |
| How often do you usually use/visit these places? | - *Many times* - *Quite Often* - *Rarely* - *Never* - *Don’t know* - *No answer provided* |
| What prevents you from enjoying these natural spaces more? | - *My physical condition/state of health limits me* - *Difficulties or lack of transport* - *My work prevents me from using these spaces more* - *The weather (extreme heat, rain, cold, etc.) prevents me from making more use of the resource* - *My family or social responsibilities limit me* - *I do not feel supported by my environment (family, friends, or others)* - *I do not feel safe or welcome in these natural spaces* - *The spaces are not clean/well maintained* - *I prefer to do other things* - *Other (specify)* - *Don’t know* - *No answer provided* |
| ***[Cultural capital]***  Traditions and/or cultural activities help us create bonds with other people and help us develop a sense of belonging to a place. Culture is everything that a community or group of people knows and does, including its traditions, beliefs, and shared history | |
| What traditions and cultural activities do you know in your neighbourhood? | - *Yes* - *No* - *No answer provided*   *If the participant mentions any, the interviewer marks ‘Yes’* |
| How often do you usually participate in these traditions and cultural activities? | - *Many times* - *Quite Often* - *Rarely* - *Never* - *Don’t know* - *No answer provided* |
| What prevents you from participating more in the traditions and cultural activities of your neighbourhood? | - *My physical condition/state of health limits me* - *I have difficulties with the language (or culture* - *Difficulties or lack of transport* - *My work prevents me from participating in these activities* - *The weather (extreme heat, rain, cold, etc.) prevents me from participating more in these activities* - *The price/cost is too high for me* - *My family or social responsibilities limit me* - *I do not feel supported by my environment (family, friends or others* - *I do not feel safe or welcome in these activities* - *The activities seem to be only for certain people, a closed group* - *There is not enough information about these activities* - *I prefer to do other things* - *Other (specify* - *Don’t know* - *No answer provided* |
| ***[Human capital]*** | |
| By putting your knowledge or skills into practice, do you think you can improve your health? | - *Yes* - *No* - *No answer provided* - *Don’t know*   *If the participant mentions any, the interviewer marks ‘Yes’* |
| Do you participate in courses, workshops or other activities to learn new things and improve your skills? | - *Yes* - *No* - *No answer provided*   *If the participant mentions any, the interviewer marks ‘Yes’* |
| How often do you participate in these courses, workshops or activities? | - *Many times* - *Quite Often* - *Rarely* - *Never* - *Don’t know* - *No answer provided* |
| What prevents you from accessing more opportunities for learning and personal development? | - *My physical condition/state of health limits me* - *I have difficulties with the language (or culture)* - *Difficulties or lack of transport* - *My job limits me* - *Inconvenient schedules* - *The price/cost is too high for me* - *My family or social responsibilities limit me* - *I do not feel supported by my environment (family, friends or others)* - *I do not feel safe* - *The activities seem to be only for certain people, a closed group* - *There is not enough information* - *I do not feel motivated* - *I prefer to do other things* - *Other (specify)* - *Don’t know* - *No answer provided* |
| ***[Social capital]*** | |
| Do you know of any community groups or organisations (such as social clubs, sports clubs, neighbourhood associations, senior citizens'/young people's associations, religious groups, hobby groups, etc.)? | - *Yes* - *No* - *No answer provided*   *If the participant mentions any, the interviewer marks ‘Yes’* |
| How often do you participate in these groups or organisations? | - *Many times* - *Quite Often* - *Rarely* - *Never* - *Don’t know* - *No answer provided* |
| What prevents you from participating more in community activities or local groups? | - *My physical condition/state of health limits me* - *I have difficulties with the language (or culture)* - *Difficulties or lack of transport* - *My work prevents me from participating in these activities* - *Inconvenient schedules* - *The weather (extreme heat, rain, cold...) prevents me from participating more in these activities* - *The price/cost is too high for me* - *My family or social responsibilities limit me* - *I do not feel supported by my environment (family, friends or others)* - *I do not feel safe or welcome in these spaces/activities* - *The spaces/activities seem to be only for certain people, a closed group* - *There is not enough information* - *I am not interested* - *Other (specify)* - *Don’t know* - *No answer provided* |
| ***[Built capital]***  The infrastructures in our community -such as sports centers, social centers, study rooms, and similar facilities- provide spaces that allow us to promote and engage in different activities that directly or indirectly influence our health | |
| What infrastructure are you familiar with? | - *Yes* - *No* - *No answer provided*   *If the participant mentions any, the interviewer marks ‘Yes’* |
| How often do you usually use these facilities? | - *Many times* - *Quite Often* - *Rarely* - *Never* - *Don’t know* - *No answer provided* |
| What prevents you from using these spaces more? | - *My physical condition/state of health limits me* - *I have difficulties with the language (or culture)* - *Difficulties or lack of transport* - *My work prevents me from participating in these activities* - *Inconvenient schedules* - *The weather (extreme heat, rain, cold...) prevents me from participating more in these activities* - *The price/cost is too high for me* - *My family or social responsibilities limit me* - *I do not feel supported by my environment (family, friends or others)* - *I do not feel safe or welcome in these spaces/activities* - *The spaces/activities seem to be only for certain people, a closed group* - *There is not enough information* - *The spaces are not clean/well maintained* - *I prefer to do other things* - *Other (specify)* - *Don’t know* - *No answer provided* |
| ***[Financial capital]*** | |
| Are you aware of any financial assistance and/or individual subsidies available to individuals or families in your neighbourhood or municipality? | - *Yes* - *No* - *No answer provided*   *If the participant mentions any, the interviewer marks ‘Yes’* |
| Would accessing this type of assistance be... | - *Very easy* - *Quite easy* - *Somewhat easy* - *Not easy at all* - *I haven't tried or haven't been asked to* - *Other (specify)* - *Don’t know* - *No answer provided* |
| What prevents you from accessing these resources? | - *Due to my personal circumstances, I would not be eligible for financial assistance, or I do not meet the requirements* - *I have difficulties with the language (or culture)* - *Difficulties or lack of transport* - *My job prevents me from accessing these resources* - *Inconvenient schedules* - *The price/cost is too high for me* - *My family or social responsibilities limit me* - *I do not feel supported by my environment (family, friends or others)* - *I do not feel safe using these resources* - *The resources seem to be only for certain people* - *There is not enough information about these resources* - *Complicated procedures* - *I do not meet the requirements* - *My physical condition/health limits my access to these resources* - *I am not interested/ Other (specify)* - *Don’t know* - *No answer provided* |
| Could you tell me about any financial assistance and/or community subsidies available for developing activities in your neighbourhood or municipality? | - *Yes* - *No* - *No answer provided*   *If the participant mentions any, the interviewer marks ‘Yes’* |
| Would accessing this type of assistance be… | - *Very easy* - *Quite easy* - *Somewhat easy* - *Not easy at all* - *I haven't tried or haven't been asked to* - *Other (specify)* - *Don’t know* - *No answer provided* |
| What prevents you from accessing these resources? | - *Due to my personal circumstances, I would not be eligible for financial assistance, or I do not meet the requirements* - *I have difficulties with the language (or culture)* - *Difficulties or lack of transport* - *My job prevents me from accessing these resources* - *Inconvenient schedules* - *The price/cost is too high for me* - *My family or social responsibilities limit me* - *I do not feel supported by my environment (family, friends or others)* - *I do not feel safe using these resources* - *The resources seem to be only for certain people* - *There is not enough information about these resources* - *Complicated procedures* - *I do not meet the requirements* - *My physical condition/health limits my access to these resources* - *I am not interested/ Other (specify)* - *Don’t know* - *No answer provided* |
| ***[Political capital]***  Political capital is the influence and power that people have to make positive changes in their community | |
| What mechanisms of influence or influential people in decision-making are you aware of in your neighbourhood or municipality? | - *Yes* - *No* - *No answer provided*   *If the participant mentions any, the interviewer marks ‘Yes’* |
| Do you feel you have the opportunity to express your opinions on issues that matter to you? | - *Many times* - *Quite Often* - *Rarely* - *Never* - *Don’t know* - *No answer provided* |
| How often do you participate in these influence mechanisms? | - *Many times* - *Quite Often* - *Rarely* - *Never* - *Don’t know* - *No answer provided* |
| What prevents you from participating more in decisions that affect your community? | - *My physical condition/health limits me* - *I have difficulties with the language (or culture)* - *Difficulties or lack of transport* - *My job limits me* - *Inconvenient schedules* - *My family or social responsibilities limit me* - *I do not feel supported by my environment (family, friends or others)* - *I do not feel safe* - *Influencing mechanisms only seem to work for certain people, closed group* - *There is insufficient information on how to participate in these mechanisms* - *I do not know how to do it* - *I don't feel listened to* - *I am not interested* - *Other (specify)* - *Don’t know* - *No answer provided* |

## **Table S6.** Questionnaire module on social capital and community cohesion (3,4)

| **Social capital: Assistance** | |
| --- | --- |
| Do you know anyone in your life who can assist you free of charge with the following kinds of situations? Do not include people whom you might hire, but only those who would help you for free | |
| - Can babysit for you in an emergency - Can lend you money if you need it (for example, at least €500) - Write a good reference/recommendation for a landlord - Can write a good reference/recommendation letter when you are applying for a job - Provide advice about local schools - Provide advice about preparing income taxes - Can help you with small jobs around the house (e.g. painting, home maintenance) - Can give you a ride (airport, mall) if you need it - Can give advice on matters of law (e.g. problems with landlord, boss) - Can do your shopping when you (and your household) are ill | *Answer options for each situation:*   - Yes^1^ - No - Not applicable |
| ^1^When yes is selected, the participants are asked to specify who the person is. Response options: Acquaintance/friend, Workplace supervisor, Workplace coworker, Family member/relative, Church member/Religious services member, Neighbour, Other. | |
| **Social capital: Interaction /Bridging** | |
| In these community or local group activities, how often do you interact with colleagues who...? | |
| - Are of a different nationality? - Speak a different language? - Are of a different race or ethnicity? - Have different political views to yours? - Have a different level of education to yours? - Are from another generation? | *Answer options for each group:*   - *Never* - Rarely - Quite often - Very often - Don’t know - No answer provided |
| **Community cohesion and sense of belonging** | |
| How would you describe your sense of belonging to Carrús^1^? | - Very strong - Quite strong - Not very strong - Not strong at all - Don’t know - No answer provided |
| How long have you lived in Carrús^1^? | - Less than 6 months - 6 months to less than 1 year - 1 year to less than 3 years - 3 years to less than 5 years - 5 years to less than 10 years - 10 years or more - Don’t know - No answer provided |
| Would you say you trust many, quite a few, few, or none of the people in Carrús^1^? | - Many people - Quite a few people - Few people - No one - Don’t know - No answer provided |
| Would you say that Carrús^1^ is a place where neighbours help each other…? | - A lot - Quite a lot - A little - Not at all - Don’t know - No answer provided |
| How many people in Carrús^1^ do you know well enough to ask for a favour? Some examples of favours are: collecting the mail, watering the plants, borrowing things, carrying items upstairs, feeding the pets, or picking up a child after school | - None - 1 or 2 - 3 to 5 - 6 or more - Don’t know - No answer provided |
| Compared to other areas of Elche, do you think there is a higher, equal, or lower level of crime in Carrús^1^? | - Higher - Equal - Lower - Don’t know - No answer provided |
| How safe do you feel from crime when walking alone in Carrús^1^ at dusk? | - Very safe - Quite safe - Not very safe - Not at all safe - Don’t know - No answer provided |
| Considering the level of crime in the neighbourhood, how safe is it for children to play outside in Carrús^1^ during the day? | - Very safe - Quite safe - Not very safe - Not at all safe - Don’t know - No answer provided |

^1^*Carrús is the name of the neighbourhood where the study is taking place.*

## **Table S7:** Questionnaire module on knowledge, attitudes and practices on personal antibiotics. (5)

| - Antibiotics are effective against viruses - When I get a cold, I take antibiotics to help me feel better faster - If I feel better after a few days, I sometimes stop taking my antibiotics before completing the course of treatment - I expect my doctor to prescribe antibiotics if I suffer from common cold or flu symptoms - It is good to be able to get antibiotics from relatives or friends without having to see a medical doctor - When I have a sore throat, I prefer to use an antibiotic - Each type of infection needs a different antibiotic - ﻿If I feel side effects during a course of treatment of antibiotics, I should stop taking them as soon as possible - ﻿I take the antibiotics according to the doctor’s instructions - ﻿If antibiotics are consumed in excess, they will not work when they are really needed - ﻿I prefer to keep antibiotics at home in case there is a need for them later - ﻿I trust the doctor’s decision if s/he decides to prescribe or not prescribe antibiotics - ﻿If I believe that I need an antibiotic and the doctor did not prescribe it, I will get it at the pharmacy without a prescription - ﻿Doctors often explain clearly to the patient the reasons for prescribing or not prescribing antibiotics - ﻿Doctors often explain clearly to the patient the instructions for the use of antibiotics - ﻿When you buy antibiotics, the pharmacist tells you about the importance of correct therapeutic compliance/adherence | *Answer options for each group:*   - Likert scale from 0 (strongly disagree) to 10 (strongly agree) |
| --- | --- |
| Have you taken antibiotics in the last two months? | - Yes - No - Don’t know - No answer provided |
| How long did your last course of antibiotics last? | - Number of days: - Number of months: |
| Who prescribed or recommended the use of antibiotics? | - The doctor - A friend - A family member - The pharmacist - No one, I had it at home - Other, please specify - Don’t know - No answer provided |
| The last time you had to take antibiotics, did you finish/complete the course? | - Yes - No - I am still taking them - Don’t know - No answer provided |
| What did you do with the antibiotics you did not use? | - I took them to the pharmacy. - I kept them to use the next time I got ill. - I threw them in the bin or down the drain/toilet/sewer. - I gave them to someone else. - I did not have any antibiotics left. - Don’t know - No answer provided |
| The last time you had to take antibiotics, did you forget to take any doses? | - I did not forget to take any doses. - I forgot to take some doses. - I forgot to take quite a few doses. - Don’t know - No answer provided |
| What did you do when you missed a dose of antibiotics? | - I did nothing and continued with the next doses when it was time. - I took 2 doses at the next dose. - I took it as soon as I remembered. - Don’t know - No answer provided |
| The last time you had to take antibiotics, did you change the dose on your own (without medical advice)? | - Yes - No - Don’t know - No answer provided |
| What did you do when you changed the antibiotic dose on your own (without medical advice)? | - I took more. - I took less. - Sometimes I took more and sometimes I took less. - Don’t know - No answer provided |
| Why did you increase the antibiotic dose on your own (without medical advice)? | - I forgot to take the previous dose. - I felt very unwell. - I didn't notice any noticeable improvement and decided to increase the dose. - I felt better, but I wanted to improve even more. |
| Why did you reduce the antibiotic dose on your own (without medical advice)? | - I was concerned about the side effects. - I forgot because I was tired. - I forgot because I was in a hurry or because of work. - I felt I was taking too many medicines. - Other. - Don’t know - No answer provided |

**References:**

1. Selim AJ, Rothendler JA, Qian SX, Bailey HM, Kazis LE. The History and Applications of the Veterans RAND 12-Item Health Survey (VR-12). J Ambulatory Care Manage. 2022 Sept 1;45(3):161–70.

2. Chinn D, McCarthy C. All Aspects of Health Literacy Scale (AAHLS): Developing a tool to measure functional, communicative and critical health literacy in primary healthcare settings. Patient Educ Couns. 2013 Feb;90(2):247–53.

3. Villalonga-Olives E, Adams I, Kawachi I. The development of a bridging social capital questionnaire for use in population health research. SSM - Popul Health. 2016 Dec;2:613–22.

4. Canadian Community Health Survey [Internet]. [cited 2025 Nov 16]. Available from: https://www.canada.ca/en/health-canada/services/food-nutrition/food-nutrition-surveillance/health-nutrition-surveys/canadian-community-health-survey-cchs.html

5. Mallah N, Rodríguez-Cano R, Figueiras A, Takkouche B. Development and validation of a knowledge, attitude and practice questionnaire of personal use of tranquilizers. Drug Alcohol Depend. 2021 July 1;224:108730.
